# A unique insight into the defect structures of bicontinuous mesophases in liquid crystals and hybrid materials

**DOI:** 10.1107/S2052252520002535

**Published:** 2020-02-29

**Authors:** Alfonso Garcia-Bennett

**Affiliations:** a Department of Molecular Sciences, Macquarie University, Sydney, NSW 2109, Australia

**Keywords:** twinning defects, electron crystallography, bicontinuous cubic structures, constant mean curvature surfaces, inorganic porous solids, crystal distortions

## Abstract

Han *et al.* [(2020), *IUCrJ*, **7**, 228–237] using advanced electron microscopy and crystallographic modelling rationalise the microstructure of twinning defects in order to visualize mesophase transitions and surface properties of G and D bicontinuous cubic mesostructured silica. This work furthers our understanding of how these phases originate in many natural and synthetic systems.

Bicontinuous structures represent nature’s intrinsic beauty and mathematical elegance in equal measures. Examples of their unique function to partition space exist in all walks of science. These range from the inner workings of cellular organelles (Deng *et al.*, 1999 ▸), to the synthesis three-dimensional photonic crystals using butterfly wings as biotemplates (Mille *et al.*, 2011 ▸) and to the engineering of lightweight material components for advanced mechanical application in orthopaedic implants (Maskery *et al.*, 2018 ▸). Bicontinuous mesophases and their biological implications were first uncovered by Luzzati and co-workers during their studies of lipid–water systems (Luzzati *et al.*, 1960 ▸). They are particularly prominent in amphiphilic surfactant systems between hexagonal and lamellar mesophases. Here they form continuous bilayers as opposed to discrete micelles such as spherical geometries (Luzzati *et al.*, 2004 ▸). Mesophase transitions are commonly rationalized by considering the *packing parameter* (*g*) of the self-assembling micellar unit, *g* = *V*/*a* *l*. This is a geometric relation involving the volume (*V*) occupied by the amphiphilic molecule divided by its cross-sectional area (*a*) and its hydro­phobic alkyl chain length (*l*) (Israelachvili, 2011 ▸). At a given packing parameter, small variations in the total apolar fraction of the amphiphilic molecule can cause phase transformation between the different cubic bicontinuous phases, from 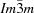 space-group symmetry to 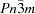 and to 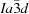 (P- to D- to G-minimal surface topology) (Fogden & Hyde, 1999 ▸).

Whilst many have followed and modelled transitions of bicontinuous cubic mesosphases in water systems using a myriad of techniques, including small-angle X-ray scattering (SAXS), NMR, calorimetric analysis, optical textures *etc*., none of these bring us closer to understanding the microstructural changes and topological defects that occur at the interface between one bicontinuous phase and another. How is the space between the two minimal surfaces partitioned? How is a minimal surface frustrated by defects? How stable are the intermediate mesophases that arise as a result? These are the questions that Han *et al.* attempt to answer in this issue of **IUCrJ** (Han *et al.*, 2020 ▸).

The approach of Han *et al.* relies on a detailed study by state-of-the-art high-resolution transmission electron microscopy and electron crystallography of bicontinuous mesoporous silica crystals, which enable the relevant bicontinuous mesophase to be frozen in a stable silica replica of the structure. The mesoporous crystals were prepared to produce particles rich in contact reflection twin defects, possessing a twin boundary plane, which separates the two identical crystalline domains (Fig. 1 ▸).

Two bicontinuous cubic phases, the G and D phases, and their corresponding G-twin and D-twin boundaries are structurally assessed revealing a detailed model of the connectivity of the surface and its curvature fluctuations at the twin position itself, as well as 3D reconstructions of the silica network. Han *et al.* describe how the formation of G-twin boundaries in the {211} plane may be related to structural transformations between hexagonal and lamellar phases. These may give rise to the G structure, supported by observed epitaxial {211} relationships between the structures involved and the generation of the essential chiral surface of the G-twin boundary. The authors also treat the D-twin as a stacking fault, which is neatly used to postulate potential stacking orders and new surface and channel motives that may arise through twinning defects in bicontinuous minimal surfaces.

This work opens a new avenues to study defect formation and changes to the surface geometry of bicontinuous minimal surfaces, and will be especially useful if it can be applied to analogous biological systems. Knowledge of the structural transformations may also help in directing the design and application of mesoporous silica particles.

## Figures and Tables

**Figure 1:**
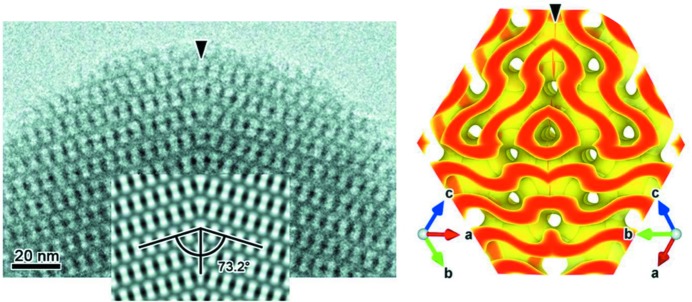
HRTEM image of bincontinous cubic (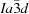) mesoporous silica crystal showing a {211} twin boundary (left) and 3D reconstructed models of the silica network of the G-twin boundary. Adapted from (Han *et al.*, 2020 ▸).
